# Prevalence and histopathological characterization of Masai Giraffe (*Giraffa camelopardalis tippelskirchi*) skin disease in Tarangire-Manyara ecosystem, Northern Tanzania

**DOI:** 10.1080/01652176.2021.1970279

**Published:** 2021-08-26

**Authors:** Faraja E. Kiula, Eblate E. Mjingo, Alex R. Mremi, Jaffu O. Chilongola, Linus K. Munishi

**Affiliations:** a The Nelson Mandela-African Institution of Science and Technology, Arusha, Tanzania; b Tanzania Wildlife Research Institute, Arusha, Tanzania; c Kilimanjaro Christian Medical Center, Moshi, Tanzania; d Kilimanjaro Christian Medical University College, Moshi, Tanzania

**Keywords:** Masai Giraffe, *Giraffa camelopardalis tippelskirchi;* giraffe skin disease, prevalence, mycosis

## Abstract

**Background:**

Masai Giraffes have declined dramatically in recent decades due to loss of habitat and illegal hunting. Hence, it is critically important that the epidemiology and etiology of so-called giraffe skin disease (GSD) is understood well.

**Aim:**

To assess the prevalence and histopathological characteristics of GSD in the Tarangire-Manyara Ecosystem (TME), northern Tanzania.

**Methods:**

The study used road transects to gather field information on GSD. Eighty-four giraffes were sighted by systematic random sampling in the six study sites. Examination of giraffes involved body distribution of lesions, severity of the lesions and whether they were associated with age and sex of the affected giraffes. Five giraffes with GSD were immobilized for tissue collection and histopathological analysis.

**Results:**

Prevalence among adults was 79%. Affected animals typically had 1–5 lesions which were mostly moderate and were predominantly observed on the forelegs. GSD positivity rate was higher among females versus males, whereas males had a higher rate of severe lesions and generally had more lesions than females. Calves showed no lesions. All tissue sections from five affected giraffes showed the presence of large quantities of fungal elements (hyphae and spores) that involved hair shafts and sub-cutaneous tissue after staining with Grocott Methenamine Silver as special fungal staining technique.

**Conclusions:**

Our findings suggest the involvement of fungal infection in GSD pathogenesis.

**Clinical relevance:**

We recommend further characterization of the lesions using modern molecular techniques and culture to identify primary and secondary or opportunistic etiologies, and the order in which the pathogens occur in the lesions.

## 1. Introduction

Tanzania supports an impressive variety of large mammal populations both in protected and outside fully protected areas, mainly in semi-arid rangelands (Stoner et al. 2007). Despite the numerous strategies implemented to enhance wildlife conservation, there is rampant population decline of large mammal species including giraffes (Stoner et al. 2007). Masai Giraffes (*Giraffa camelopardalis tippelskirchi*) have declined dramatically in recent decades due to loss of habitat and illegal hunting, and perhaps diseases (Stoner et al. 2007; Okello et al. 2015). A skin disease known as giraffe skin disease (GSD) was first described in Ruaha National Park, Central Tanzania in 2000, in which about 80–85% of the giraffe population with 92% of adults affected (Mpanduji et al. 2011; Epaphras et al. 2012). GSD has also been reported as occurring in the Tarangire-Manyara Ecosystem (TME) and Serengeti-Ngorongoro Ecosystem, but not in Arusha National Park (Lee and Bond, 2016). GSD occurrence in other areas of Tanzania is unknown. Although it was speculated that GSD may negatively impact giraffe populations (Mpanduji et al. 2011; Epaphras et al. 2012), capture-recapture analysis of adults in the TME found no difference in survival probabilities or reproduction associated with presence or severity of GSD lesions (Whittier et al. 2020), making GSD a less serious population-level threat (Lee and Bond, 2016). In order to implement feasible control strategies against the spread of GSD (Mpanduji et al. 2011), it is critically important that its epidemiology and etiology is understood.

The only previous study that attempted to identify the causative agent of GSD had inconclusively reported the involvement of a spirurid nematode as a potential etiology of GSD. Although bacterial and fungal elements were observed in the study, they were both considered as secondary invaders (Mpanduji et al. 2011). Nonetheless, none of these etiologies has however been confirmed as the primary cause of GSD. In the absence of conclusive results on the causative agent of GSD, we hypothesize that, in addition to what is known regarding the gross features of GSD, understanding the histopathological features of GSD will provide better insights into the identity of a potential etiology of GSD. The overall aim of this study was to examine the prevalence and describe histopathological features of GSD lesions in attempts to get insights into the pathogenesis and possible etiology of GSD.

## 2. Methods

### 2.1. Study sites

**Figure 1.**
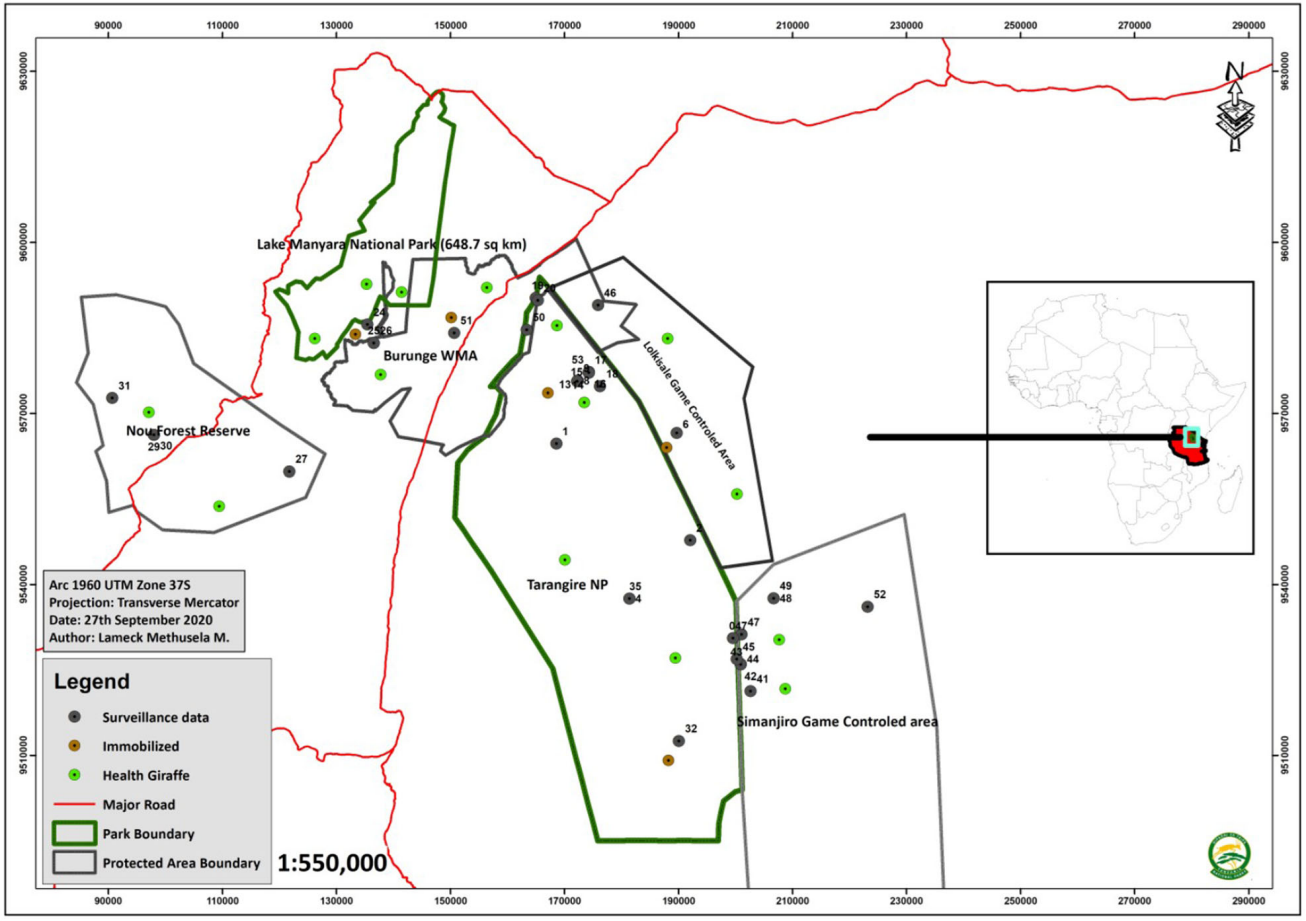
Map of Tarangire-Manyara ecosystem in Northern Tanzania showing study transects with distribution of giraffes observed during the field survey.

The study was carried out in the TME in northern Tanzania at six sites representing various types of conservation areas. Study sites included Tarangire National Park (TNP), Lake Manyara National Park and protected areas such as Burunge Wildlife Management Area (Burunge WMA), Lolksale Game Controlled Area (Lolksale GCA), Nou Forest Reserve and Simanjiro Game Reserve (Simanjiro GR) (Figure 1).

### 2.2. Assessment of prevalence of giraffe skin disease

A total of 84 giraffes in 16 groups were sighted and observed in 6 sites over a span of forty days from February to April, 2019. The surveys were done early in the morning between 8.00 and 11.00 am and in the evening between 5.00 and 6.00 pm when it was cool and giraffes were actively browsing. Two weeks (14 days) were used to select giraffe with clear signs of GSD for immobilization and biopsy collection. The transects were laid down based on potential areas inhabited by giraffes and accessibility of protected areas road network. Two researchers were equipped with binoculars and hand-held global positioning system (GPS) units, sitting on the middle sit of the car observing each side of the transect. The car was driven on maximum speed of 20 km/h and whenever an individual or group of giraffes was seen, the car was stopped for observation of each individual for the presence of GSD. In order to minimize potential bias of double sighting of giraffes, a systematic random selection of study animals was adopted. On each day, out of the first 10 giraffes encountered, every 5th giraffe was randomly selected for examination. In cases the 5th giraffe was not clearly visible, the 6th giraffe was examined. Parameters taken into consideration included size of the group, sign of giraffe skin disease, location, number and distribution of the lesions on the body, age and sex of the affected individual.

### 2.3. Definition of GSD lesion severity and age categories

GSD lesions were categorized into four categories based on severity. These categories were grouped as (i) asymptomatic (ii) mild lesion (initial stages of nodules of < 5 cm diameter, (iii) moderate lesion (between 5 and 10 cm of diameter, and (iv) severe lesions with a diameter ≥11 cm (Figure 3). Age classes were categorized as calves (<12 months), sub adult (12 months to < 4 years), or adult (4+ years). Calves which stay with their mother’s show folded or wrinkled skins, large eyes and ears relative to the face. Sub adults have smoother skins with small ossicones having black hair at their tips. While the tall adult giraffes have tight skin on the face and jaw areas, darkening coat color with mane waving (Strauss 2013).

### 2.4. Giraffe immobilization procedures

Five giraffes with clearly visible sign of GSD on fore limbs, hind limbs and brisket areas and the lesions ranging from mild, moderate to severe were immobilized at a distance of about 30 m to 40 m. Immobilization was performed in areas with scarce vegetation and appropriate terrain. Etorphine hydrochloride (M99) (Wildlife Pharmaceuticals, Mpumalanga, South Africa) was used at total dose of 20–25 mg per animal using a dart gun (Cap-chur®, Palmer Company, Powder Springs, GA, USA). Immobilization was terminated by intravenous injection of a reversing agent (antidote) Diprinophine (M5050) (Wildlife Pharmaceuticals, Mpumalanga, South Africa) at 72 mg/kg BW. To boost the cardio-pulmonary performances, inotropes and respiratory stimulants were given at the time of recovery.

### 2.5. Sample collection and histopathological processing of skin biopsies

One skin biopsy was taken from GSD lesions of each of the five immobilized giraffes by using the punch method as described previously (Zuber 2002). Briefly, the most affected area or abnormal-appearing sites or the edge of an actively growing lesion of GSD were selected for biopsy. The area to be biopsied was cleansed with povidone-iodine solution. The punch biopsy instrument with a diameter of 2 mm was held perpendicular to the surface of the lesion. The instrument was pressed down into the lesion while it was being rotated clockwise and counter-clockwise, cutting down into the subcutaneous fat. The punch biopsy instrument was removed and the biopsy specimen gently lifted with a needle to avoid crush artifact. Scissors were used to cut the specimen free at a level below the dermis. Since the punch biopsy defects were small, no suturing was done post biopsy.

Biopsies were immersed into 10% neutral buffered formalin to prevent decomposition and stored in biohazard bags filled with dry ice at −20 °C until transported to the laboratory for histopathological analysis. By using a microtome, tissues were sectioned in thin sections of 3–5 µm thick, then placed in microscopic glass slides ready for staining. Frozen biopsies stained by Hematoxylin and Eosin (HE) as described previously by Feldman and Wolfe (Feldman and Wolfe 2014) while Grocott Methenamine Silver (GMS) (Agilent, Santa Clara, CA, USA) staining was performed according to Ma and colleagues (Ma et al. 2013). The tissue sections were oxidized in 0.5% periodic acid solution for 15 min at room temperature, rinsed three times in distilled water, and incubated in methenamine silver working solution for 30 min to 1 h at 60 °C. Sections of the tissue were rinsed in hot distilled water, checked microscopically, and then rinsed in distilled water at room temperature and toned in gold chloride solution for 1 min, rinsed in distilled water, treated with sodium thiosulfate solution for 2 min, and then washed in running tap water for 10 min. The sections were counterstained in nuclear light green for 5 min, and then subjected to dehydration, clearing in xylene, and mounting with a coverslip.

Sections of the tissue were scanned at different magnifications to characterize sections using Olympus Light Microscope BX43F (Olympus Corp, Tokyo, Japan). Interpretation was judged by two independent pathologists with consensus. For the HE staining, different section elements were interpreted as follows: collagen (pale pink), muscle (deep pink), acidophilic cytoplasm (red), basophilic cytoplasm (purple), nuclei (blue) and erythrocytes (cherry red). Interpretation of microscopic slides stained with GMS was as follows: fungal cell wall (black/dark crown), inner parts of mycelia and hyphae (old rose), *Leishmania spp*, *Toxoplasma spp* (negative), mucin (dark grey) and background (pale green). Black or brown-darkish staining was considered as positive for fungal cell wall.

### 2.6. Data processing and analysis

Field and laboratory data was analyzed using Statistical Product and Service Solutions (IBM SPSS Armonk, NY, and USA) software version 22. Descriptive data of categorical variables was presented in the form of numbers and percentages organized into Tables. Chi square test (χ^2^) was used to determine associations between variables using a P value of 0.05 as the statistical cut-off point.

## 3. Results

Overall GSD prevalence was 58/84 (69% [Table 1]), with symptomatic animals almost entirely adults plus one sub-adult, and no calves showing lesions (Table 2). Prevalence among adults was 79%. Affected animals typically had 1–5 lesions, had mostly moderate lesions, and lesions were mostly observed on the forelegs (Table 1). GSD positivity rate was higher among females versus males (Table 2), but males had a higher rate of severe lesions and generally had more lesions than females (Table 3).

**Table 1.** Descriptive statistics for prevalence and distribution of Giraffe Skin Disease in studied giraffes.

Variable (n)		Number	Percent
Positivity for GSD (n = 84)	Negative	26	31
	Positive	58	69
Sex (n = 84)	Female	44	53
	Male	40	47
Lesion Severity (n = 58)	Mild	21	36
	Moderate	25	43
	Severe	12	21
Partly affected (n = 58)	Front Leg	42	72
	Brisket	3	5
	Hind Leg	13	23
Number of Lesions (n = 58)	1 lesion	16	28
	1 to 5 lesions	32	55
	> 5 lesions	10	17

**Table 2.** Association between Sex and Age of giraffes with GSD positivity (n = 84).

Variable	Category	GSD positivity		Total (n = 84)	χ^2^, p value
Sex		^a^Positive; n (%)	^b^Negative; n (%)		
	Female	34 (59%)	10 (39%)	44 (52%)	χ^2^ =2.93, p = 0.08
	Male	24 (41%)	16 (61%)	40 (48%)	
	Total	58 (100%)	26 (100%)	84 (100%)	
Age Group	Calves	0 (0%)	4 (15%)	4 (5%)	χ^2^ = 24.342, p = 0.000
	Sub adults	1 (2%)	7 (27%)	8 (9%)	
	Adults	57 (98%)	15 (58%)	72 (86%)	
	Total	58 (100%)	26 (100%)	84 (100%)	

^a^ Giraffes with GSD (n = 58); ^b^Giraffes without GSD (n = 26); Positive; Positivity for GSD was strongly associated with adult giraffes (χ^2^ = 24.342, p = 0.000).

**Table 3.** Distribution of GSD lesions by severity, affected parts and number of lesions across sex and age groups of giraffes.

Lesion severity						
	Age group	Category	Mild	Moderate	Severe	Totals
		Sub Adults	1 (100%)	0 (0%)	0 (0%)	1
		Adults	20 (35%)	25 (44%)	12 (21%)	57
		Total	21 (36%)	25 (43%)	12 (21%)	58
	Sex	Female	11 (32%)	17 (50%)	6 (18%)	34
		Male	10 (42%)	8 (33%)	6 (25%)	24
		Total	21 (36%)	25 (43%)	12 (21%)	58
Affected Parts						
	Age group	Category	Front Leg	Brisket	Hind Leg	Total
		Sub Adults	1 (100%)	0 (0%)	0 (0%)	1
		Adults	41 (72%)	3 (5%)	13 (23%)	57
		Total	42 (72%)	3 (5%)	13 (23%)	58
	Sex	Female	27 (79%)	0 (0%)	7 (21%)	34
		Male	15 (63%)	3 (13%)	6 (24%)	24
		Total	42 (72%)	3 (5%)	13 (23%)	58
No. of Lesions		Category	1 Lesion	1–5 Lesions	> 5 Lesions	Total
	Age group	Sub Adults	1 (100%)	0 (0%)	0 (0%)	1
		Adults	15 (26%)	32 (56%)	10 (18%)	57
		Total	16 (28%)	32 (55%)	10 (17%)	58
	Sex	Female	9 (26%)	20 (59%)	5 (15%)	34
		Male	7 (29%)	12 (50%)	5 (21%)	24
		Total	16 (28%)	32 (55%)	10 (17%)	58

All numbers and percentages are computed using a denominator of 58, the number of giraffes with GSD.

**Figure 2.**
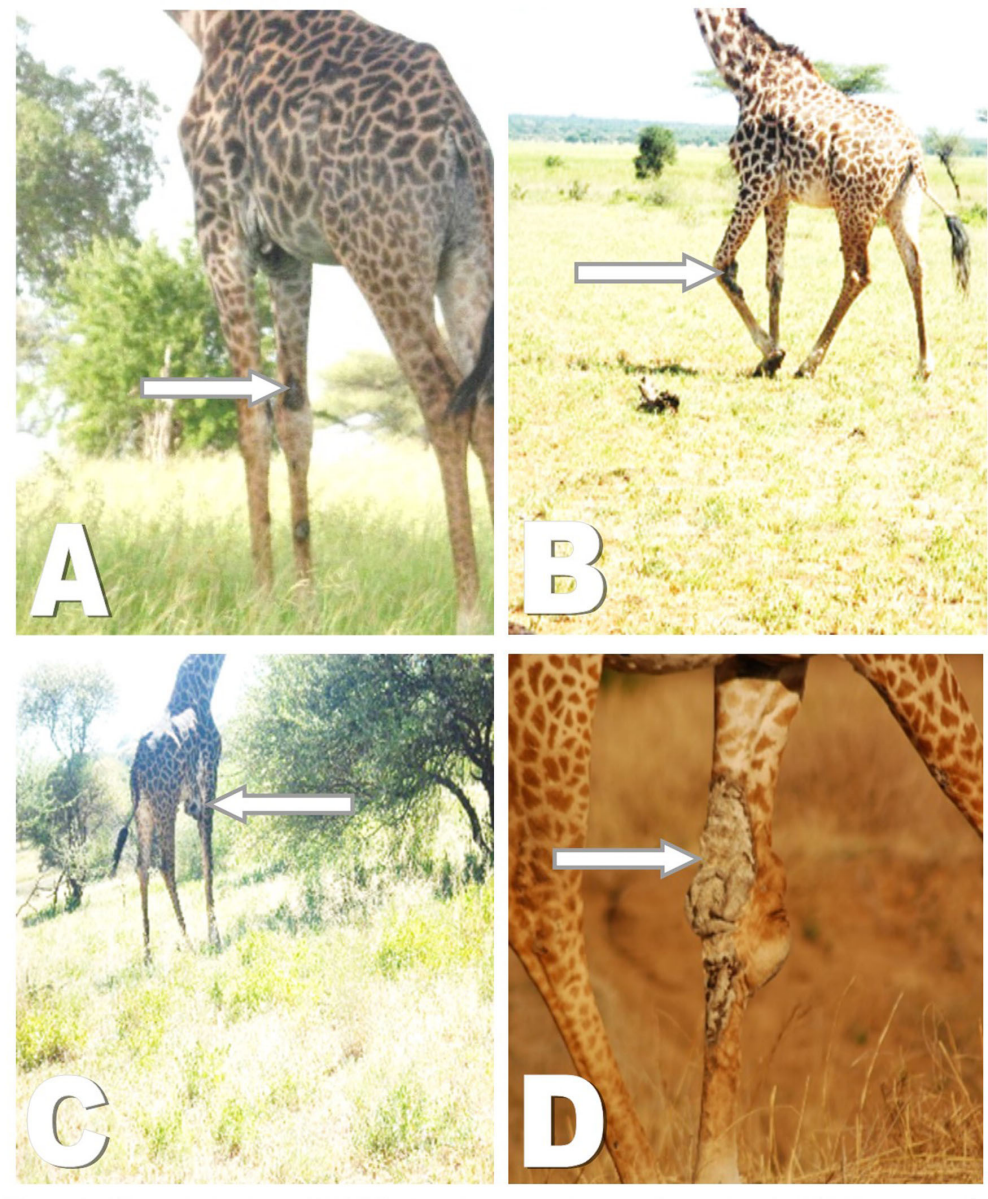
Characterization of giraffe skin disease lesions based on degree of severity. A: Mild lesion on fore limbs, B: Moderate lesions with lumpy appearance and sores C: Moderate lesions on brisket characterized by inflammation D: Severe lesion, severe wrinkling and inflammation.

The only subadult with GSD was found to have a single lesion in the forelimb (Tables 1–3). Generally, the gross lesions observed included scabs, wrinkled skin, encrustations (Figure 2) while dried or fresh oozing blood was observed on some GSD lesions. Other lesions noticed include skin flaps, pendulous skin and cracking of the skin with exudates due to presumed secondary infection. One affected animal had a noticeably swollen carpal joint.

### 3.1. Histopathological analysis of GSD tissues biopsies

**Figure 3.**
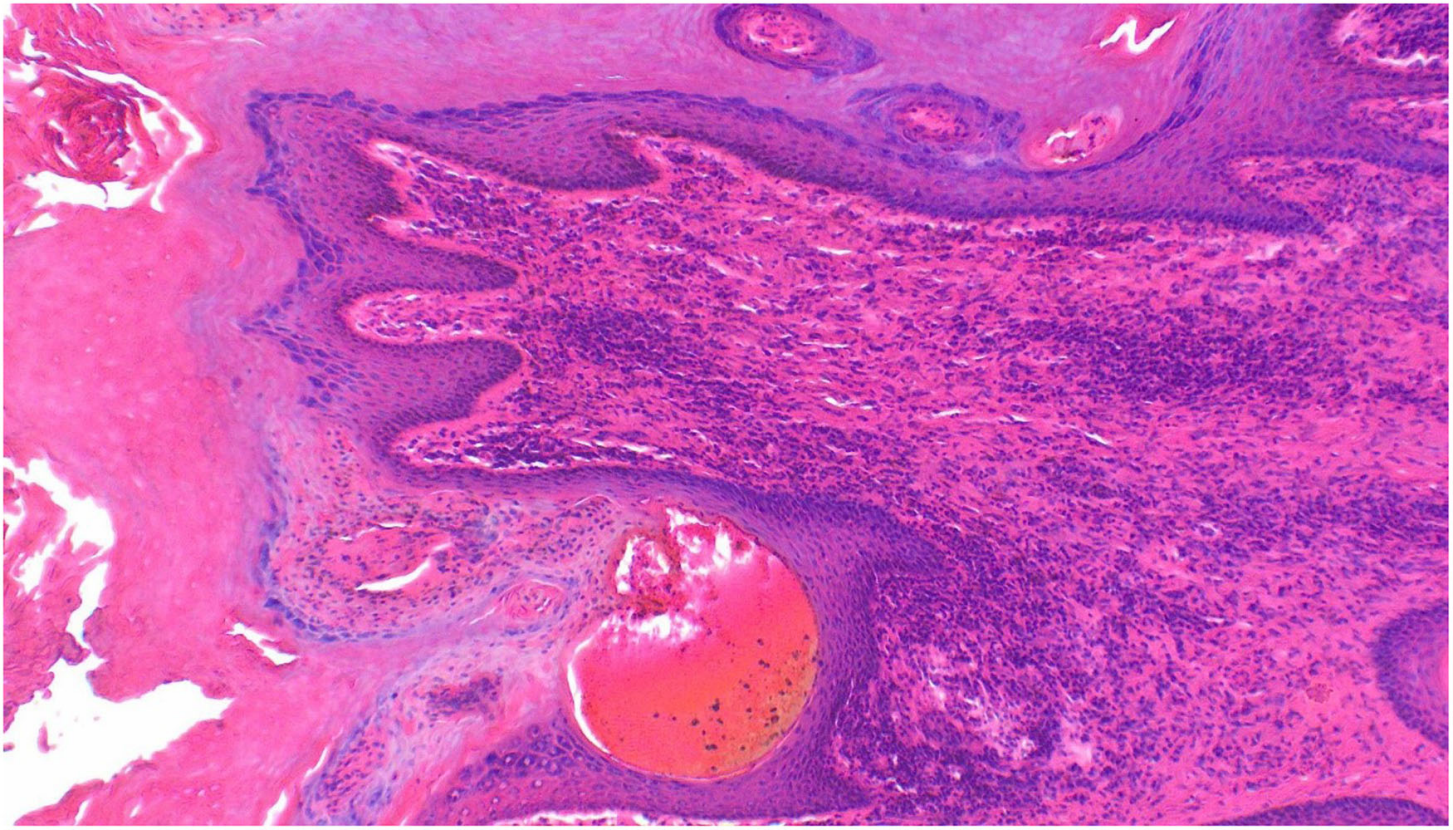
Giraffe skin disease section by HE staining showing a complex of fungal hyphae, chronic focal inflammation and extravasation of red blood cells (magnification of 10 times) [arrows].

**Figure 4.**
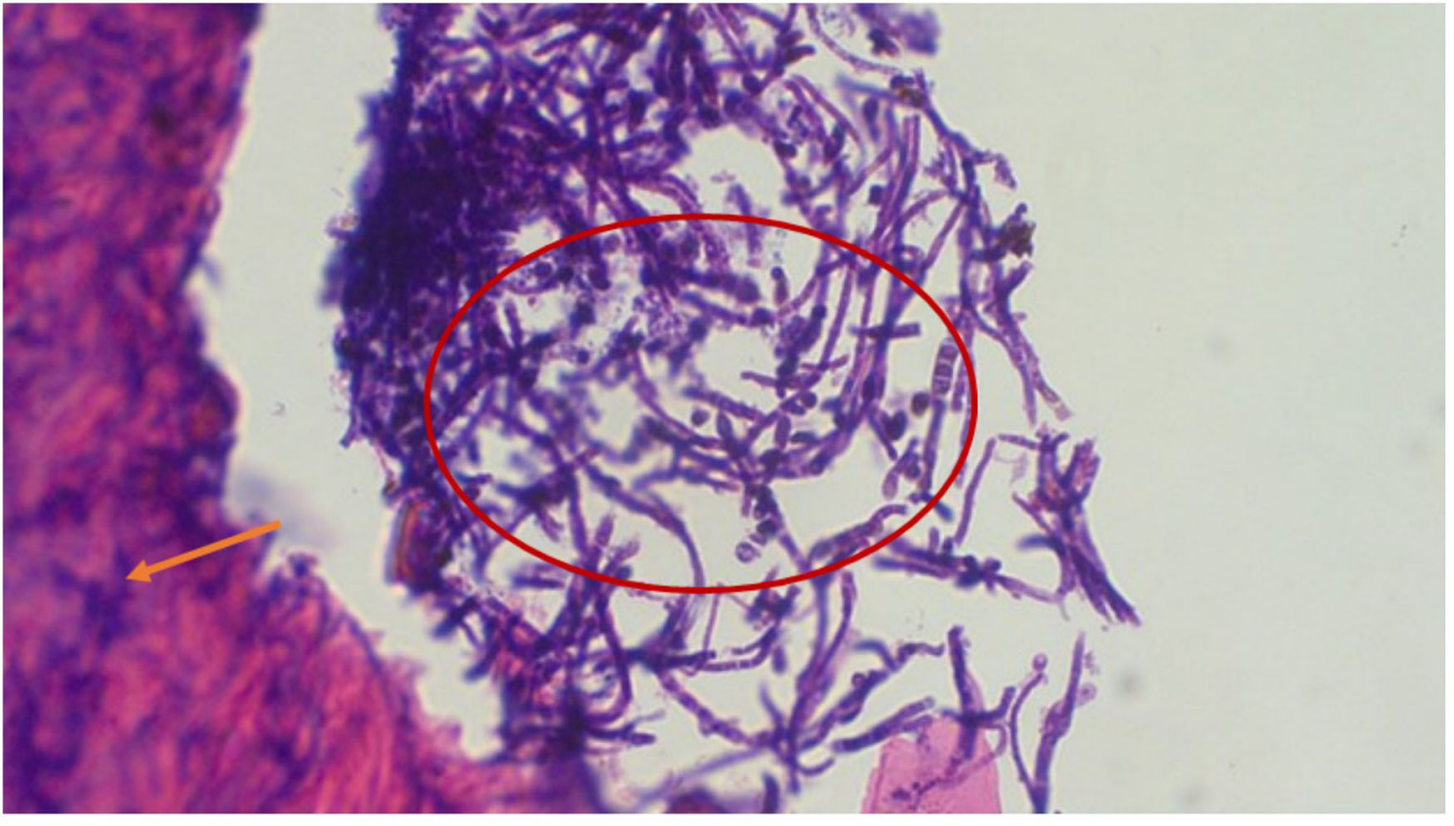
Giraffe skin disease section by HE staining showing a complex of fungal hyphae (circle) chronic inflammation and extravasation of red blood cells (magnification of 40 times) [arrows].

**Figure 5.**
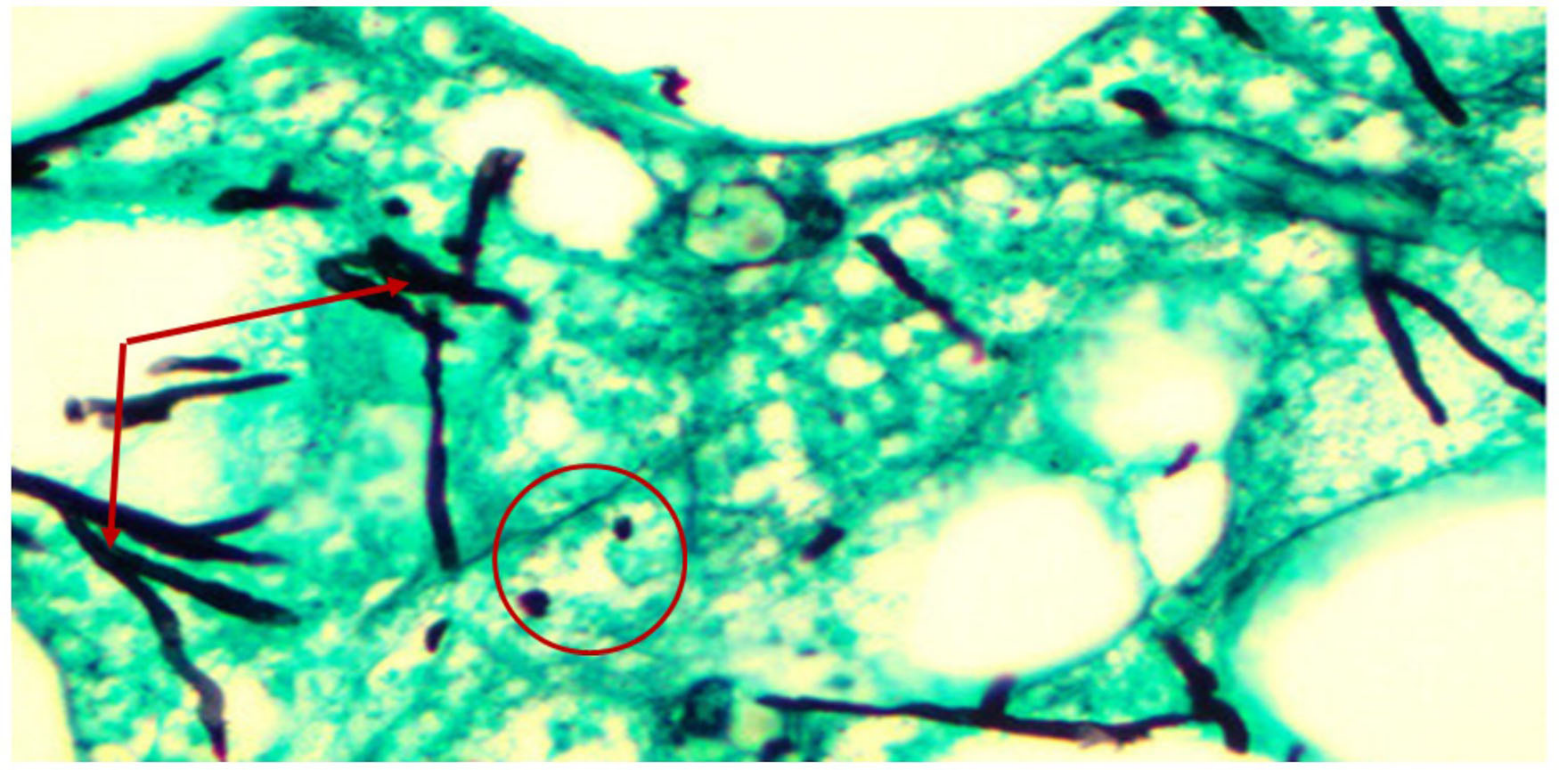
Photo microscopy of giraffe skin disease tissue section showing fungal spores (circle) and hyphae (arrows) which are positive for Grocott’s Methenamine-Silver special fungal staining (magnification of 10 times).

Biopsies from giraffes with severe GSD lesions were subjected to histopathological staining. All tissue sections from five affected giraffes showed the presence of large quantities of fungal elements (hyphae and spores) that involved hair shafts and sub-cutaneous tissue as revealed by photo microscopy (Figures 3 and 4). Grocott's methenamine silver stain (Figure 5) revealed numerous round spores with thick double walls, occurring singly or in chains connected by tubular projections. Fungi were seen as prominent black filaments of varying length with two parallel borders. Septae and branching fungal filaments were clearly identified in all 5 tissue sections. GMS special stain is also used for staining some bacteria such as *Nocardia spp*., *Mycobacterium spp.,* and non-filamentous bacteria with polysaccharide capsules, such as *Klebsiella pneumoniae* and *Streptococcus pneumoniae*. Our histopathological results have revealed none of these bacterial species. The GMS technique is not used to detect nematodes. We were therefore unable to identify any types of previously reported nematodes by this technique.

## 4. Discussion

The current study shows that GSD is highly prevalent in the Tarangire Manyara Ecosytem. The severity, distribution and number of the lesions indicated that in the TME, the severity of GSD ranged from mild to moderate with 21% of affected animals having severe lesions. This observation is contrary to what was previously reported in Ruaha national park that 52% of the animals were severely affected (Epaphras et al. 2012). Previous studies have associated soil types with occurrence of GSD (Bond et al. 2016). Differences in soil types in the different ecosystems where GSD is prevalent may be a probable reason to explain this discrepancy. In the current study there was no GSD case in calves. However, adult giraffes were most affected compared to sub adults.

Histopatological examination of GSD lesions from TME have consistently revealed the presence of fungal elements by using a special fungal staining technique. The few previous studies that characterized lesions from GSD in Ruaha National Park had implicated a nematode as the causative agent of GSD (Mpanduji et al. 2011). Analysis of biological samples from seven affected giraffes collected from a skin disease with clinical manifestations similar to GSD from Uganda revealed a parasitic worm that was likely to originate from the genus *Stephanofilaria* (Whittier et al. 2020), transmitted among domestic cattle through biting flies. Other studies have previously reported a set of bacteria species as the likely primary cause of GSD, making it challenging to draw solid conclusions from these reports (Epaphras et al. 2012; Lee and Bond, 2016). However, since it is not conclusive whether the skin diseases reported outside Tanzania (Ruaha National Park and Tarangire-Manyara Ecosystem) (Muneza et al. 2016) is actually GSD, it is still difficult to conclude the etiological agent of GSD.

This study has shown fungal infection to be highly prevalent in all samples from all 5 affected giraffes. This observation suggests a likely association between fungal infestation and GSD. Whether fungal infestation is the primary etiology or not, it remains to be confirmed by studies that will involve large numbers of giraffes from different geo-ecological regions of Tanzania and abroad. Further, studies that will adopt molecular techniques and isolation and characterization methods of isolates from the lesions will be valuable in defining the etiology of GSD.

## 5. Conclusion and recommendation

This study has demonstrated the distribution of giraffe skin disease, its spatial pattern in Tarangire-Manyara ecosystem in Northern Tanzania and differences in GSD between sexes, age, part of the body affected and histopathological features. The disease affects mainly forelimbs of adults but not caves. Fungal infection is an important component of the GSD lesions. We recommend further characterization of the isolates from lesions using modern molecular techniques to identify primary and secondary or opportunistic etiologies, and the order in which the pathogens occur in the lesions.
